# Undergraduate nursing students’ COVID-19 vaccine intentions: A national survey

**DOI:** 10.1371/journal.pone.0261669

**Published:** 2021-12-22

**Authors:** Holly B. Fontenot, Deborah B. Mattheus, Eunjung Lim, Alexandra Michel, Nicole Ryan, Amelia Knopf, Nadia N. Abuelezam, Kelly Stamp, Barbara Hekel, Sandra Branson, Gregory Zimet

**Affiliations:** 1 School of Nursing & Dental Hygiene, University of Hawaii at Manoa, Honolulu, Hawaii, United States of America; 2 Department of Quantitative Health Sciences, John A. Burns School of Medicine, University of Hawaii at Manoa, Honolulu, Hawaii, United States of America; 3 Department of Community and Health Services, School of Nursing, Indiana University, Indianapolis, Indiana, United States of America; 4 Connell School of Nursing, Boston College, Chestnut Hill, Massachusetts, United States of America; 5 School of Nursing, University of North Carolina at Greensboro, Greensboro, North Carolina, United States of America; 6 Cizik School of Nursing, UTHealth, Houston, Texas, United States of America; 7 Division of Adolescent Medicine, School of Medicine, Indiana University, Indianapolis, Indiana, United States of America; Bay Area Hospital, North Bend Medical Center, UNITED STATES

## Abstract

**Introduction:**

In December 2020, the first two COVID-19 vaccines were approved in the United States (U.S.) and recommended for distribution to front-line personnel, including nurses. Nursing students are being prepared to fill critical gaps in the health care workforce and have played important supportive roles during the current pandemic. Research has focused on vaccine intentions of current health care providers and less is known about students’ intentions to vaccinate for COVID-19.

**Methods:**

A national sample of undergraduate nursing students were recruited across five nursing schools in five U.S. regions in December 2020. The survey measured perceived risk/threat of COVID-19, COVID-19 vaccine attitudes, perceived safety and efficacy of COVID-19 vaccines, sources for vaccine information and level of intention to become vaccinated [primary, secondary (i.e., delayed), or no intention to vaccinate].

**Results:**

The final sample consisted of 772 students. The majority (83.6%) had intentions to be vaccinated, however of those 31.1% indicated secondary intention, a delay in intention or increased hesitancy). The strongest predictors of primary intention were positive attitudes (OR = 6.86; CI = 4.39–10.72), having lower safety concerns (OR = 0.26; CI = 0.18–0.36), and consulting social media as a source of information (OR = 1.56; CI = 1.23–1.97). Asian (OR = 0.47; CI = 0.23–0.97) and Black (OR 0.26; CI = 0.08–0.80) students were more likely to indicate secondary intention as compared to primary intention. Students in the Midwest were most likely to indicate no intention as compared to secondary intention (OR = 4.6; CI = 1.32–16.11).

**Conclusions:**

As the first two COVID-19 vaccines were approved/recommended in the U.S. nursing students had overall high intentions to vaccinate. Findings can guide development of educational interventions that reduce concerns of vaccine safety that are delivered in a way that is supportive and affirming to minoritized populations while being respectful of geo-political differences.

## Introduction

At the time of this study, December 2020, two vaccines that provide protection against coronavirus disease 2019 (COVID-19) (mRNA BNT162B2 vaccine, developed by BioNtech and Pfizer, and the mRNA-1273 vaccine, developed by Moderna) had recently received United States (U.S.) Food and Drug Administration (FDA) Emergency Use Authorization (EUA) [1, 2]. Due to elevated risk for exposure/acquisition, the Advisory Committee on Immunization Practices (ACIP)/Center of Disease Control and Prevention (CDC) recommended that health care providers be offered vaccination in the primary phase of vaccine distribution [3, 4]. Nurses, in particular, are at the greatest risk for COVID-19 infection due to prolonged exposure during direct patient care [5]. In the spring of 2020, of all health care providers hospitalized, over one-third were in nursing related occupations [6].

Despite this elevated risk, published reports conducted during vaccine development (prior to December 2020) have highlighted a wide range of vaccine intention among U.S. nurses, ranging from 34–60% indicating they would obtain the vaccine as it became available [7, 8], with many nurses remaining uncertain (31%) [7]. Low COVID-19 vaccine intention could be related to general vaccine hesitancy, which has been noted as a growing concern in the U.S. and abroad [9–11]. National media coverage of COVID-19 vaccine development as ‘rushed’, reflected in the U.S. government’s use of the term ‘warp speed’, may have contributed to unwarranted suspicion and mistrust in the vaccine development process, resulting in the potential for low confidence in hypothetical COVID-19 vaccine(s) [12].

Ongoing examinations of COVID-19 vaccine intentions and vaccine uptake among nurses are important to inform continued interventions supporting vaccine decision making for the profession as well as the public at large. Additionally, the COVID-19 pandemic may be accelerating the rate of nurse retirement [13, 14], which poses a serious risk for a national nursing shortage during a critical time in public health [15]. Vaccine availability and uptake may resolve pandemic related nursing workforce drop out; however, even before the pandemic, national data indicated a growing rate of aging nurses likely heading for retirement [16, 17]. The nursing profession will rely on students currently enrolled in school to stabilize the workforce. Therefore, it is imperative to examine students’ vaccine intentions to ensure interventions support a robust future workforce during and after the pandemic.

Nursing students are being prepared to fill the inevitable gaps in the health care workforce, plus where able, they have played a critical role in the current pandemic response as students (e.g., assisting with screening, testing, and direct patient care). While most reports in the U.S. have focused on current providers, one study to-date examined nursing students’ future intentions to vaccinate at a university in the Northeast U.S. region. This study indicated that 45% of students would receive the COVID-19 vaccine once it became available [8]. Among, youth of similar age (18–29) in the general population 37% indicated they were willing to receive a COVID-19 vaccine once it became available [18].

It is critical for both future employers and nursing schools to understand COVID-19 vaccine intentions and their correlates among nursing students nationally. Findings can support interventions specifically tailored to support students joining the profession during a global pandemic. Therefore, the purpose of this study was to 1) assess intentions to obtain a COVID-19 vaccine, and 2) identify factors that are associated with vaccine intention among a national sample of undergraduate nursing students at the time of vaccine approval and recommendation. We specifically sought to explore level of vaccine intention (primary, secondary, or no intention). It is important to understand differences in intention (elucidate factors related to hesitation- secondary intention) to tailor educational interventions to convert hesitant persons to vaccinated persons as well as plan for ongoing vaccine distribution efforts overtime.

## Materials and methods

### Participants and recruitment

In December of 2020, we conducted an electronic survey of undergraduate nursing students recruited across five nursing schools in five U.S. regions (Northeast, Southeast, Southwest, Midwest, and West). Three public and two private universities were included, and enrollment ranged from 150 to 795 students per school. Data were collected during the same timeframe as the FDA EUA approval and ACIP/CDC recommendation for the first two COVID-19 vaccines, and vaccine distribution efforts had not yet started nationally. Data were collected and managed using a secure Research Electronic Data Capture (REDCap) web-based survey and database hosted at the primary investigator’s institution. Using Dillman’s Tailored Design Method [19, 20], recruitment emails were sent to the enrolled nursing students at each of the five nursing schools (approximately 2,085 recipients). A site investigator from each partner institution was responsible for sending the study’s standardized recruitment emails to their respected listservs. The emails included a link to the study webpage for further study information, informed consent procedures, and assessment of eligibility. If a potential participant was eligible and provided consent, they were directed to continue the 15-minute study survey. Participation was voluntary, and each student was offered a $5.00 electronic Starbucks gift card distributed by the study’s principal investigator. Individuals were eligible to participate if they were: 1) currently enrolled full-time as an undergraduate nursing student in one of the five partnered nursing schools, 2) 18 years of age or older, and 3) able to read and understand English. Students enrolled in graduate programs were excluded from this study. Individual schools were blinded to the rate of student participation, study data were anonymous, and participation was voluntary. All remuneration data was kept confidential and separate from study data. The University of Hawaii at Manoa Institutional Review Board (IRB) served as the primary IRB and study approval was granted on November 4, 2020.

### Sample

A total of 868 participants opened the survey, 866 provided consent, and 800 met eligibility criteria (current undergraduate nursing student). Of these, only 38 students did not respond to the vaccine intention items- the majority of these students also failed to complete most of the survey. The final sample included 772 students who completed the survey and responded to the vaccine intention items. The final sample represented approximately 37% of those invited to participate.

The majority of participants were 18–23 years (78.8%), female (87.6%), and Non-Hispanic (NH) White (58.3%), and reported the highest level of parent or primary care-giver as college graduate or more (61.4%). Student year was identified as 11.6% freshman, 17.6% sophomore, 30.6% junior, 35.4% senior, and 4.8% other. Of the participants, 19.4% currently attended a nursing school in the Northeast, 11.5% in the Southeast, 26.0% in the Southwest, 35.6% in the Midwest, and 7.4% in the West (socio-demographic characteristics are presented in Table 1). Among participants, 52.5% indicated primary intention (n = 405), 31.1% secondary intention or hesitancy (n = 240), and 16.4% no intention (n = 127) to receive the COVID-19 vaccine (patterns of vaccine intention by socio-demographics are presented in Table 1 and patterns of intention by COVID-19 related variables are presented in Table 2).

**Table 1:** Socio-demographics, vaccine intentions, and bivariate association between socio-demographics and COVID-19 vaccine intention.

	Total	Intentions, n (%)			Bivariate Association, OR [95% CI]	
Socio-Demographics	N (%)	Primary (n = 405, 52.5)	Secondary (n = 240, 31.1)	No (n = 127, 16.4)	Primary vs. Secondary	No vs. Secondary
Age						
18–23 yrs.	608 (78.8)	321 (79.3)	194 (80.8)	93 (73.2)	1.00	1.00
24–29 yrs.	99 (12.8)	55 (13.6)	28 (11.7)	16 (12.6)	1.19 [0.73, 1.94]	0.97 [0.53, 1.80]
30+ yrs.	65 (8.4)	29 (7.2)	18 (7.5)	18 (14.2)	0.97 [0.53, 1.80]	**2.09 [1.04, 4.20]**
Sex						
Male/Other	96 (12.4)	56 (13.8)	28 (11.7).	12 (9.4)	1.00	1.00
Female	676 (87.6)	349 (86.2)	212 (88.3)	115 (90.6)	0.82 [0.51, 1.34]	1.27 [0.62, 2.58]
Race/Ethnicity						
NH White	450 (58.3)	253 (62.5)	124 (51.7)	73 (57.5)	1.00	1.00
Hispanic	105 (13.6)	53 (13.1)	39 (16.3)	13 (10.2)	0.67 [0.42, 1.06]	0.57 [0.28, 1.13]
NH Asian	131 (17.0)	73 (18.0)	45 (18.8)	13 (10.2)	0.80 [0.52, 1.22]	**0.49 [0.25, 0.97]**
NH Black	44 (5.7)	10 (2.5)	19 (7.9)	15 (11.8)	**0.26 [0.12, 0.57]**	1.34 [0.64, 2.80]
NH NHOPI/Mixed/ Other	42 (5.4)	16 (4.0)	13 (5.4)	13 (10.2)	0.60 [0.28, 1.29]	1.70 [0.75, 3.86]
Student Year						
Freshman	87 (11.6)	52 (13.2)	23 (9.8)	12 (9.8)	1.00	1.00
Sophomore	132 (17.6)	74 (18.8)	39 (16.6)	19 (15.4)	0.84 [0.45, 1.57]	0.93 [0.38, 2.27]
Junior	230 (30.6)	119 (30.3)	74 (31.5)	37 (30.1)	0.71 [0.40, 1.26]	0.96 [0.43, 2.14]
Senior	266 (35.4)	129 (32.8)	87 (37.)	50 (40.7)	0.66 [0.37, 1.15]	1.10 [0.51, 2.40]
Other	36 (4.8)	19 (4.8)	12 (5.1)	5 (4.1)	0.70 [0.29, 1.68]	0.80 [0.23, 2.80]
Highest Education Level of Parent or Primary Care Giver						
≤ Some College	288 (38.6)	129 (33.0)	111 (47.6)	48 (39.0)	1.00	1.00
≥ College Graduate	459 (61.4)	262 (67.0)	122 (52.4)	75 (61.0)	**1.85 [1.33, 2.58]**	1.42 [0.91, 2.22]
Having Family Member as Healthcare Provider	306 (40.9)	161 (41.0)	88 (37.8)	57 (46.7)	1.14 [0.82, 1.60]	1.45 [0.93, 2.25]
Employed as Healthcare Worker	272 (36.4)	143 (36.5)	83 (35.6)	46 (37.7)	1.04 [0.74, 1.46]	1.09 [0.70, 1.72]
Political View						
Liberal	304 (40.7)	208 (53.1)	79 (33.9)	17 (13.9)	**2.08 [1.41, 3.07]**	**0.42 [0.22, 0.79]**
Neither	231 (30.9)	105 (26.8)	83 (35.6)	43 (35.2)	1.00	1.00
Conservative	124 (16.6)	42 (10.7)	44 (18.9)	38 (31.1)	0.76 [0.45, 1.26]	1.67 [0.94, 2.95]
Prefer not to answer	88 (11.8)	37 (9.4)	27 (11.6)	24 (19.7)	1.08 [0.61, 1.92]	1.72 [0.89, 3.33]
State						
Northeast (Massachusetts)	150 (19.4)	103 (25.4)	40 (16.7)	7 (5.5)	1.00	1.00
West (Hawaii)	57 (7.4)	35 (8.6)	16 (6.7)	6 (4.7)	0.85 [0.42, 1.70]	2.14 [0.62, 7.37]
Midwest (Indiana)	275 (35.6)	131 (32.3)	85 (35.4)	59 (46.5)	**0.60 [0.38, 0.94]**	**3.97 [1.66, 9.46]**
Southeast (North Carolina)	89 (11.5)	42 (10.4)	27 (11.3)	20 (15.7)	0.60 [0.33, 1.11]	**4.23 [1.57, 11.39]**
Southwest (Texas)	201 (26.0)	94 (23.2)	72 (30.0)	35 (27.6)	**0.51 [0.32, 0.82]**	**2.78 [1.13, 6.82]**

OR = Odds Ratio. CI = Confidence Interval. NH = Non-Hispanic. Column percentages were computed. Bivariate association was explored using a multinomial logistic regression model with each variable as a predictor and vaccine intention as the outcome variable, treating secondary intention as the reference.

**Table 2:** COVID-related predictors, vaccine intentions, and bivariate association between COVID-19 related predictors and COVID-19 vaccine intention.

	Total	Intentions, n(%)/ mean ± SD			Bivariate Association	
COVID-19 Related Predictors	N (%)/ mean ± SD	Primary (n = 405, 52.5)	Secondary (n = 240, 31.1)	No (n = 127, 16.4)	Primary vs. Secondary	No vs. Secondary
Tested for COVID-19						
No	238 (30.8)	120 (29.6)	71 (29.6)	47 (37.0)	1.00	1.00
Yes	534 (69.2)	285 (70.4)	169 (70.4)	80 (63.0)	1.00 [0.70, 1.42]	0.72 [0.45, 1.13]
The Result of COVID-19 Test ^a^						
Tested Positive	45 (8.4)	22 (7.7)	12 (7.1)	11 (13.8)		
Tested Negative	484 (90.6)	262 (91.9)	155 (91.7)	67 (83.8)		
Still Awaiting the Results	5 (0.9)	1 (0.4)	2 (1.2)	2 (2.5)		
Health concern make infection more severe						
No	655 (84.8)	341 (84.2)	202 (84.2)	112 (88.2)	1.00	1.00
Yes	117 (15.2)	64 (15.8)	38 (15.8)	15 (11.8)	1.00 [0.64, 1.55]	0.71 [0.38, 1.35]
Likelihood of Infection	29 (3.8)	4 (1.0)	6 (2.5)	19 (15.0)	0.39 [0.11, 1.39]	**6.86 [2.67, 17.67]**
Had a family member or close friend become infected with COVID-19						
No	301 (39.0)	162 (40.0)	96 (40.0)	43 (33.9)	1.00	1.00
Yes	471 (61.0)	243 (60.0)	144 (60.0)	84 (66.1)	1.00 [0.72, 1.39]	1.30 [0.83, 2.04]
Think COVID-19 infection is a major problem in your home community						
No	271 (35.1)	128 (31.6)	69 (28.8)	74 (58.3)	1.00	1.00
Yes	501 (64.9)	277 (68.4)	171 (71.3)	53 (41.7)	0.87 [0.62, 1.24]	**0.29 [0.18, 0.45]**
Think COVID-19 infection is a major problem in your school community?						
No	303 (39.2)	139 (34.3)	89 (37.1)	75 (59.1)	1.00	1.00
Yes	469 (60.8)	266 (65.7)	151 (62.9)	52 (40.9)	1.13 [0.81, 1.57]	**0.41 [0.26, 0.64]**
Mean Perceived Severity (2 items)	2.1 ± 0.9	2.1 ± 0.9	2.3 ± 1.0	1.9 ± 0.9	0.85 [0.72, 1.00]	**0.60 [0.47, 0.78]**
Mean Worry (3 items)	3.6 ± 1.2	3.8 ± 1.0	3.6 ± 1.1	2.9 ± 1.4	1.15 [0.99, 1.33]	**0.62 [0.52, 0.74]**
Mean General COVID-19 Vaccine Attitude (2 items)	4.0 ± 1.0	4.6 ± 0.5	3.7 ± 0.7	2.4 ± 1.0	**8.42 [6.02, 11.78]**	**0.16 [0.10, 0.23]**
Mean Lack of Perceived Safety (3 items)	3.2 ± 1.0	2.7 ± 0.9	3.7 ± 0.7	4.2 ± 0.8	**0.22 [0.17, 0.29]** ***	**2.74 [1.99, 3.76]** ***
Effectiveness of Vaccine	3.3 ± 0.8	3.3 ± 0.8	3.3 ± 0.8	3.4 ± 0.9	0.94 [0.78, 1.14]	1.23 [0.93, 1.63]
Trustworthy Information: Social Media	2.1 ± 1.1	2.2 ± 1.2	2.0 ± 1.0	2.0 ± 1.2	**1.18 [1.02, 1.36]**	1.00 [0.82, 1.22]
Trustworthy Information: Other Sources (e.g., healthy agency, CDC) (7 items)	3.9 ± 0.7	4.1 ± 0.6	3.8 ± 0.6	3.4 ± 0.8	**2.13 [1.62, 2.80]**	**0.52 [0.38, 0.71]**

OR = Odds Ratio. CI = Confidence Interval. SD = Standard Deviation ^a^Among those who received COVID-19 test. Measures were averaged over items, indicating 1 = Strongly disagree to 5 = Strongly agree. Effectiveness of Vaccine is Likert 4-type scale: 1 = Not at all, 2 = Not very much, 3 = Some, 4 = A great deal. Likelihood of Infection, Likelihood of getting a COVID-19 vaccine if recommended, and Likelihood of getting a COVID-19 vaccine if required were Likert 5-type items: 1 = Strongly disagree or Very unlikely to 5 = Strongly agree or Very likely. Column percentages or means (and SDs) were computed. Bivariate association was explored using a multinomial logistic regression model with each variable as a predictor and vaccine intention as the outcome variable, treating secondary intention as the reference.

*P<0.05.

**P<0.01.

***P<0.001.

### Measures

The study team collected socio-demographic information and data on students’ intention to vaccinate, personal beliefs regarding risk/threat of COVID-19 infection, COVID-19 vaccine attitudes, perceived safety and efficacy, and trusted sources of COVID-19 vaccine information. For this survey, the study team adapted scales previously reported as valid and reliable [21].

#### Socio-demographics

Age, gender, race/ethnicity, student year, current employment in healthcare, political view, and state where attending university were collected. We also assessed highest education level of the respondent’s parent/primary caregiver and if any family members were employed as healthcare providers. Age was categorized as 18–23 years, 24–29 years, and 30 years or older. Based on its distribution, race/ethnicity was categorized as Non-Hispanic (NH) White, Hispanic, NH Asian, NH Black, or Other. Other included Native Hawaiian and other Pacific Islanders (NHOPI), American Indian or Alaska Native (AI/AN), and individuals of multiple races. Student year was categorized as freshman (1^st^ year of their undergraduate university education), sophomore (2^nd^ year), junior (3^rd^ year), senior (4^th^ year), and other (e.g., students who fall outside the traditional categorization of grades). The highest education level of the parent/primary caregiver was categorized as two groups: 1) some college or lower and 2) college graduate or more. Having a family member who is a healthcare provider and one who is employed in healthcare were both defined as dummy variables (1 = Yes, 0 = No). Political view was measured with a 5-point Likert item (very liberal, somewhat liberal, moderate/middle-of-the-road, somewhat conservative, very conservative) with a choice for ‘prefer not to answer.’ Based on its distribution, political view was categorized as liberal, moderate, conservative, and prefer not to answer. U.S. region was identified by the state in which the participant was enrolled in school.

#### Vaccine intention

Vaccine intention was defined by the following two questions: “Will you get a COVID-19 vaccine as soon as it becomes available/offered to you?” and “Would you get a COVID-19 vaccine within a year after it became available/was offered to you?” These two questions were combined and categorized into three groups: 1) primary intention (the student intends to vaccinate as soon as able); 2) secondary intention (the student intends to vaccinate within a year after vaccine availability), and 3) no intention (the student responded ‘no’ to both questions).

#### Risk/threat of COVID-19 infection

To measure personal beliefs related to COVID-19 risk and threat, we used two scales (perceived worry and severity) and asked several single item questions. COVID-19 related worry was measured by three 5-point Likert items (1 = strongly disagree to 5 = strongly agree): “I am scared about getting COVID-19,” “The possibility of getting infected in the future with COVID-19 concerns me,” and “I don’t really worry about getting COVID-19” (reverse coded). In this study, this measure demonstrated excellent reliability (Cronbach’s alpha = 0.90). These items were averaged (mean scores across scale items) to evaluate overall COVID-19 related worry.

Three 5-point Likert items were used to measure perceived severity of COVID-19: “I am afraid that I may die if I contract COVID-19 (or if I contract COVID-19 again),” “I am at greater risk of dying if I contract COVID-19 because of my general health,” and “If you got COVID-19, how threatening would it be to your physical health?” This measure showed good reliability in this study (Cronbach’s alpha = 0.84). These items were averaged to evaluate overall perceived severity of COVID-19.

Participants also responded to the following single item questions: 1) if they had ever been tested for COVID-19, and what those results were; 2) if they had a health concern that would make infection with COVID-19 more severe; 3) if they had a family member or close friend who had been infected with COVID-19; and 4) if they thought COVID-19 was a major problem for their home and/or school community.

#### COVID-19 vaccine attitudes/perceived safety

Two scales measured vaccine attitudes and lack of perceived safety. General COVID-19 vaccine attitudes were measured by two 5-point Likert items (1 = Strongly disagree to 5 = Strongly agree). The two items were “The COVID-19 vaccine will be important for my health” and “Getting a COVID-19 vaccine will be important for the health of others in my community.” This measure demonstrated good reliability (Cronbach’s alpha = 0.86). These items were averaged to evaluate overall COVID-19 vaccine attitudes.

Lack of perceived safety was measured using three items: “I am concerned that the COVID-19 vaccine has not been around long enough to be sure it is safe,” “I am concerned about serious side effects of the COVID-19 vaccine,” and “I am concerned that the COVID-19 vaccine might cause lasting health problems for me.” These measures showed good reliability (Cronbach’s alpha = 0.86) and were averaged to evaluate lack of perceived safety.

#### Effectiveness of COVID-19 vaccine

Effectiveness of vaccine was defined by the following 4-point Likert item: “If you were making a decision about getting the COVID-19 vaccine, how much would effectiveness of the vaccine influence your decision?” (1 = Not at all, 2 = Not very much, 3 = Some, 4 = A great deal).

#### Sources for COVID-19 vaccine information

Trust in a source for COVID-19 vaccine information was assessed using a 5-point Likert item (1 = Strongly disagree to 5 = Strongly agree): “When you are looking for trustworthy information about COVID-19, how often do you consult any of following resources?” The resources were: 1) nursing school professors; 2) peer-reviewed/database information (e.g., professional journals/PubMed/Up-to-date/CINHAL); 3) professional healthcare organizations; 4) local public health agencies (e.g., city or county websites); 5) national public health agencies (e.g., CDC website); 6) social media; 7) hospital-based websites; and 8) your personal healthcare provider. Principal component factor analysis, a data reduction method, identified two factors: social media and all other sources. Social media was used as a single item question. The other seven source items were averaged to measure ‘other information’. The seven items of the ‘other information’ presented good reliability (Cronbach’s alpha = 0.82).

### Statistical analysis

All analyses were conducted in SAS 9.4 (SAS Institute, Cary NC), and *p* ≤ 0.05 was considered statistically significant. The sample was described using frequencies and percentages or means and standard deviations, depending on the type of variable. Bivariate association between vaccine intention and a socio-demographic characteristic or a predictor was examined using a univariable multinomial logistic regression on vaccine intention with secondary vaccine intention as the reference for the outcome variable. Any bivariate association of *p* < 0.15 was included in subsequent analyses. More traditional levels such as 0.05 can fail in identifying variables known to be important [22, 23]. Then we conducted a two-step hierarchical multivariable multinomial logistic regression analysis. In Step 1, we entered as a block socio-demographic variables which are relatively immutable and as a result are not ideal for intervention. In Step 2, we then added COVID-19 related behavioral and attitudinal variables which address characteristics that maybe amenable to intervention. Odds ratios (OR) and 95% confidence intervals (CI) were computed to evaluate association with vaccine intention.

## Results

### Bivariate analysis

In bivariate analyses, the socio-demographic characteristics, age, race/ethnicity, highest education level of parent or primary caregiver, political view, and U.S. region were significantly associated with vaccine intention (see Table 1 for bivariate analysis of socio-demographic characteristics and vaccine intention). Additionally, among the COVID-19 related predictors, perceived severity, worry, likelihood of infection, thinking that COVID-19 infection is a major problem in home and/or school community, general COVID-19 vaccine attitude, lack of perceived safety, and trustworthy source of information–social media and/or other sources were significantly associated with vaccine intention (see Table 2 for bivariate analysis of COVID-19 related variables and vaccine intention).

### Multivariate analysis

Hierarchical multinomial logistic regressions were conducted with variables that had bivariate associations with *p* < 0.15 (Table 3). In Step 1, race/ethnicity, highest education level of parent/primary caregiver, political view, and state were significantly associated with the intention variable. Compared to NH White, those who identified as NH Black were less likely to indicate primary vaccine intention than secondary intention (OR = 0.23, 95% CI = 0.10–0.52). Students with parents of college graduate or more education level were more likely to indicate primary intention than secondary intention (OR = 1.61, 95% CI = 1.13–2.30) as compared to students with parents who obtained some college or less. Students having liberal political views had 1.98 times higher odds of primary intention than students who selected neither/neutral political views (95% CI = 1.32–2.96).

**Table 3:** Multivariable multinomial logistic regression.

	Step 1: Socio-Demographics^#^		Step 2: Socio-Demographics + Covid-19 Related Predictors	
Variable	Primary vs. Secondary	No vs. Secondary	Primary vs. Secondary	No vs. Secondary
Age				
18–23 yrs.	1.00	1.00	1.00	1.00
24–29 yrs.	1.13 [0.67, 1.92]	0.91 [0.45, 1.85]	1.40 [0.67, 2.92]	0.83 [0.32, 2.12]
30+ yrs.	1.37 [0.70, 2.69]	1.53 [0.71, 3.28]	1.69 [0.70, 4.13]	1.81 [0.62, 5.25]
Race/Ethnicity				
NH White	1.00	1.00	1.00	1.00
Hispanic	0.71 [0.42, 1.22]	0.65 [0.30, 1.40]	0.84 [0.42, 1.69]	1.31 [0.43, 4.01]
NH Asian	0.67 [0.39, 1.12]	0.70 [0.31, 1.55]	**0.47 [0.23, 0.97]**	2.17 [0.71, 6.66]
NH Black	**0.23 [0.10, 0.52]**	1.43 [0.63, 3.24]	**0.26 [0.08, 0.80]**	1.66 [0.55, 5.04]
NH NHOPI/Mixed/ Other	0.34 [0.10, 1.15]	1.90 [0.61, 5.92]	0.64 [0.10, 4.06]	0.71 [0.15, 3.33]
Highest Education Level of Parent or Primary Care Giver				
Some college or less	1.00	1.00	1.00	1.00
College graduate or more	**1.61 [1.13, 2.30]**	1.51 [0.94, 2.43]	1.22 [0.76, 1.96]	1.24 [0.64, 2.39]
Political View				
Liberal	**1.98 [1.32, 2.96]**	**0.39 [0.20, 0.75]**	1.30 [0.76, 2.25]	0.54 [0.24, 1.23]
Neither	1.00	1.00	1.00	1.00
Conservative	0.66 [0.39, 1.13]	1.35 [0.74, 2.37]	0.54 [0.25, 1.13]	0.60 [0.25, 1.46]
Prefer not to answer	1.26 [0.69, 2.28]	1.50 [0.75, 3.00]	0.92 [0.41, 2.08]	0.70 [0.27, 1.80]
U.S. Region				
Northeast	1.00	1.00	1.00	1.00
West	1.18 [0.54, 2.58]	1.84 [0.49, 6.99]	1.16 [0.40, 3.33]	2.75 [0.54, 14.06]
Midwest	0.66 [0.39, 1.09]	**3.17 [1.27, 7.92]**	0.98 [0.47, 2.05]	**4.60 [1.32, 16.11]**
Southeast	0.72 [0.37, 1.40]	**3.08 [1.08, 8.78]**	1.12 [0.45, 2.77]	3.55 [0.83, 15.19]
Southwest	0.71 [0.41, 1.23]	2.36 [0.90, 6.20]	0.97 [0.45, 2.10]	2.59 [0.70, 9.51]
Mean Perceived Severity (2 items)			0.98 [0.72, 1.32]	0.84 [0.55, 1.30]
Mean Worry (3 items)			0.91 [0.68, 1.21]	0.93 [0.64, 1.34]
Likelihood of Infection			0.49 [0.08, 2.98]	2.94 [0.80, 10.78]
Think COVID-19 infection is a major problem in your home community				
No			1.00	1.00
Yes			0.69 [0.41, 1.18]	0.68 [0.32, 1.42]
Think COVID-19 infection is a major problem in your school community?				
No			1.00	1.00
Yes			1.13 [0.67, 1.90]	0.80 [0.37, 1.72]
Mean General COVID-19 Vaccine Attitude (2 items)			**6.86 [4.39, 10.72]**	**0.16 [0.10, 0.27]**
Mean Lack of Perceived Safety (3 items)			**0.26 [0.18, 0.36]**	**2.86 [1.81, 4.52]**
Effectiveness of Vaccine			1.13 [0.82, 1.55]	1.04 [0.69, 1.59]
Trustworthy Information: Social Media			**1.56 [1.23, 1.97]**	1.18 [0.85, 1.63]
Trustworthy Information: Other Sources (e.g., healthy agency, CDC) (7 items)			1.01 [0.66, 1.54]	1.02 [0.62, 1.70]

OR = Odds Ratio. CI = Confidence Interval. NH = Non-Hispanic. NHOPI = Native Hawaiian or Pacific Islander.

Mean perceived severity, worry, general COVID-19 vaccine attitude, and lack of perceived safety were scored to indicate 1 = strongly disagree to 5 = strongly agree. Multivariable multinomial logistic regressions were conducted with each variable as a predictor and vaccine intention as the outcome variable, treating secondary intention as the reference. *P<0.05. **P<0.01. ***P<0.001. ^**#**^The variables in the shaded area were not used in the model.

Comparing no intention to secondary intention, no significant differences were found in socio-demographics except political view and U.S. region. Students having liberal political views were less likely to have no intention to vaccinate than secondary intention to vaccinate (OR = 0.39, 95% CI = 0.20–0.75). Compared to those who attended university in the Northeast, students studying in the Midwest and Southeast had respectively 3.17 and 3.08 times the odds of no intention to vaccinate than secondary intention to vaccinate.

In Step 2, we found significant associations of vaccine intention with race, U.S. region, general COVID-19 vaccine attitude, lack of perceived safety, and consulting social media for trustworthy information (see Table 3). Compared to NH White, NH Asian and NH Black students were less likely to indicate primary intention than secondary intention to vaccinate (NH Asian: OR = 0.47, 95% CI = 0.23–0.97; NH Black: OR = 0.26, 95% CI = 0.08–0.80). As compared to students in the Northeast, students studying in the Midwest had 4.60 times the odds of indicating no intention to vaccinate compared to indicating a secondary intention to vaccinate (95% CI = 1.32–16.11). For every one unit increase in general vaccine attitude, the odds for indicating primary intention were 6.86 times greater compared to those who had a secondary intention (95% CI = 4.39–10.72). The odds for indicating no intention were decreased by a factor of 0.16 (95% CI = 0.10–0.27) for every one unit increase in general vaccine attitude. The odds for indicating primary intention over secondary intention decreased by a factor of 0.26 (95% CI = 0.18–0.36) for every one unit increase in lack of perceived safety, but for those indicating no intention over secondary intention odds increased by a factor of 2.86 (95% CI = 1.81–4.52). Students who consulted social media as a trustworthy source of information had 1.56 times greater odds of indicating primary intention than secondary intention (95% CI = 1.23–1.97).

## Discussion

This study was conducted during the same time frame as the FDA EUA and CDC/ACIP recommendation for the first two COVID-19 vaccines. Our data indicate that nursing students have overall positive attitudes towards COVID-19 vaccination and cumulatively high intentions (83.6%) to be vaccinated. Over 50% of students planned on becoming vaccinated as soon as it was offered to them (primary intention) and over 30% planned on being vaccinated within the year (secondary intention). Less than 20% had no intention to be vaccinated. The strongest predictors of primary intention were positive attitudes towards vaccination, lower concerns related to vaccine safety, and, interestingly, consulting social media as a trusted source of information. Students identifying as NH Asian and NH Black were more likely to indicate secondary intention as compared to primary intention to vaccinate- highlighting a degree of hesitancy among racial minority groups as compared to students who identified as White. Lastly, students in the Midwest were most likely to indicate no intention to vaccinate than any other U.S. region that we examined.

Having favorable attitudes towards COVID-19 vaccination and lower concerns related to COVID-19 vaccine safety increased the odds of primary intention. These findings are consistent with numerous other national and international reports [24–27]. This suggests that messaging about the COVID-19 vaccine should continue to emphasize safety data, and ongoing vaccine interventions should focus on improving attitudes towards vaccination.

Our findings with regard to racial/ethnic differences are also consistent with data from the general population. A long history of mistreatment of racial and ethnic minority groups in health care and health research has undermined trust in research and medicine [27–30]. This is concerning as racial/ethnic minorities have an increased risk of infection, severity of infection, and death from COVID-19 [31–33]. Distrust likely drives both low participation in COVID-19 vaccine research and low uptake of a vaccine. It may take a long time to repair trust, but to start, support and funding are needed for community leadership and community-based participatory action research to uncover and address unique concerns among diverse and marginalized populations.

Multiple reports have highlighted the association between political leanings and intention to be vaccinated, not only for the COVID-19 vaccine but vaccines in general [29, 34–36]. There has been a growing mistrust of vaccine science [10–12] fueled, in 2020, by increased political polarization and the linking of political identity to COVID-19 prevention measures, including vaccination [37]. Students who identified as liberal were nearly 2 times more likely to indicate primary intention to vaccinate. Additionally, based on geo-political maps, both the South and Midwest tend to have higher rates of politically conservative viewpoints [38], which may explain the regional patterns of intention in this study. Reiter et al. (2020) similarly reported differences in COVID-19 vaccine intention by U.S. geographic region [39]. More research is needed to understand this association between locality and vaccine intention to respectfully tailor public health campaigns for various communities [37].

About 80% of people in the U.S. receive their news on a digital device, 50% use social media regularly, and younger generations lead online engagement [40–42]. While overall trust in social media as sources of information was relatively low among the students, having higher levels of trust increased the odds of primary intention to vaccinate. Future research is needed to explore the impact of social media channels (who or what people are following) and content (positive or negative health messaging) on behavioral intentions. While much misinformation and disinformation exists online to decrease vaccine intention [43–45], it is important to examine how social media can make a positive impact on health and vaccination, particularly among youth [46].

Findings from this study should be viewed in terms of their limitations. The cross-sectional design has predictive limitations. Future longitudinal designs would be ideal to explore factors that impact vaccine intentions over time, especially as the landscape of the pandemic and vaccine science are evolving rapidly. Additionally, scales were adapted from Head et al., 2020 [21] and we were not able to confirm the psychometric properties of the various health belief scales in a nursing student sample prior to the launch of this study. However, we found that the scales demonstrated good internal reliability (coefficient alpha) and good predictive validity in the current study. This provides evidence that the components of the scale are sufficiently intercorrelated. However, the mean score of Likert scales questions may not be ideal measures. Future study should validate the measures we used or consider using an alternative method such as Rasch analysis. This study was the first to examine intentions to an actual vaccine(s), and intentions were greater (83.6%) than intentions to a theoretical vaccine (45%) in a previous report of nursing students conducted in August-September, 2020 [8]. Additionally, since this data was collected a third vaccine (an adenovirus vector vaccine developed by Janssen/Johnson & Johnson) has also received FDA EUA [47]. This vaccine has different characteristics (e.g., vector, doses, side effect profile), and future research should explore the potential impact vaccine characteristics may have on intention. While garnering perspectives nationally, the study is limited by the regional and racial/ethnic distribution in its sample. Additionally, we did not elucidate which social media platforms students utilized, who they followed on social media, or if they had been exposed to anti-vaccine messaging.

## Conclusion

This study is the first study to examine intention to vaccinate and factors associated with COVID-19 among a national sample of undergraduate nursing students. The study was novel as it uncovered vaccine intention (primary, secondary, or no intention) at the same time as the first two COVID-19 vaccines received approval and recommendation in the U.S. Findings will guide vaccine educational interventions for educational institutions and employers to support vaccine acceptance among nursing students and emerging professional nurses who were at first hesitant (secondary intention) or had no intention to vaccinate. Educational programs should focus on reducing concerns to vaccine safety and should be delivered in a way that is supportive and affirming to racial/ethnic minority groups and respectful of various political viewpoints. Future research should examine channel and content for vaccine promotion campaigns on social media targeting youth as well as track vaccine completion rates among those who at first identified as having secondary or no intention for vaccination.

## Supporting information

S1 Dataset (XLSX)

## Abbreviations

COVID-19: Coronavirus Disease 2019
U.S.: United States
CDC: Centers for Disease Control and Prevention
ACIP: Advisory Committee on Immunization Practices
ORs: odds ratios
CI: confidence intervals
M: mean
SD: standard deviation
FDA: Food and Drug Administration
EUA: Emergency Use Authorization
NH: Non-Hispanic
